# Memory for affixes in a long-lag priming paradigm

**DOI:** 10.16995/glossa.5735

**Published:** 2021-10-14

**Authors:** Phoebe Gaston, Linnaea Stockall, Sarah VanWagenen, Alec Marantz

**Affiliations:** Department of Psychological Sciences, University of Connecticut, USA; Department of Linguistics, Queen Mary University of London, United Kingdom; NYUAD Institute, New York University Abu Dhabi, United Arab Emirates; University of California Los Angeles, USA; NYUAD Institute, New York University Abu Dhabi, United Arab Emirates; Department of Psychology, New York University, USA; Department of Linguistics, New York University, USA

## Abstract

Psycholinguistic research on the processing of morphologically complex words has largely focused on debates about how/if lexical stems are recognized, stored, and retrieved. Comparatively little processing research has investigated similar issues for functional affixes. In Word or Lexeme Based Morphology (Aronoff 1994), affixes are not representational units on par with stems or roots. This view is in stark contrast to the claims of linguistic theories like Distributed Morphology (Halle & Marantz 1993), which assign rich representational content to affixes. We conducted a series of eight visual lexical decision studies, evaluating effects of derivational affix priming along with stem priming, identity priming, form priming, and semantic priming at long and short lags. We find robust and consistent affix priming (but not semantic or form priming) with lags up to 33 items, supporting the position that affixes are morphemes, i.e., representational units on par with stems. Intriguingly, we find only weaker evidence for the long-lag stem priming effect found in other studies. We interpret this potential asymmetry in terms of the salience of different morphological contexts for recollection memory.

## 1 Introduction

Language depends on the combination of discrete units. Within linguistics, theorists have debated whether these units are words or smaller, sublexical units, called “morphemes,” but the hierarchical arrangement of such discrete units forms the bedrock of linguistic analysis. Morphemes include stems, like the *work* in *worker*, and affixes, like the suffix -*er* in *worker*. As explained in Marantz (2013; 2016), morphemes are abstract types in the sense that they are reducible neither to sound (or letter strings) nor to meaning. Morphemes are the atomic units that underlie a language’s mapping between sound and meaning. As abstract types, tokens of morphemes are identical to each other, despite variation in the pronunciation or the contextualized meaning of any use of a morpheme. For example, each utterance of *cat* may differ in its acoustic properties and in its specific meaning in the sentential and discourse context in which it is uttered, but each occurrence is a repetition of the same morpheme and identical as such; each occurrence is a token of the same exact type.

We present evidence in this paper that long-lag priming, where a prime word influences the processing of a related target over many intervening words, reflects the effects of repetition of abstract morphemes. In immediate priming, where the prime and target are presented in succession, the orthographic and semantic similarity between prime and target may modulate the priming effect, even when prime and target share a morpheme and thus trigger type-identity priming. In long-lag priming, only the repetition of a morpheme unit generally seems to yield a priming effect, and form and meaning relations between prime and target are largely irrelevant. Establishing that English suffixes exhibit long-lag priming (in the absence of long-lag form and semantic priming), we argue that suffixes are combinatorial morphemes on par with stems. In addition, we elucidate the nature of long-lag priming as contrasted with immediate priming and discuss the relationship between long-lag priming and recollection memory for conceptual types.

Debates that raged through much of the 80s and 90s (see e.g. the Past Tense debate; Pinker & Ullman 2002) about whether complex words are parsed into their constituent morphemes whenever they are encountered have been largely resolved thanks to convergent evidence from a range of languages for rapid, automatic, form-based decomposition from behavioral masked-priming experiments (Longtin, Segui & Hallé 2003; Rastle & Davis 2008) and single word reading magnetoencephalography studies (Lewis, Solomyak & Marantz 2011; Neophytou et al. 2018; Wray, Stockall and Marantz 2021). However, linguistic theories and psycholinguistic models of word recognition still vary in what status they give to affixes.

Aronoff’s model (Aronoff 1994) is perhaps the most prominent linguistic model in which a clear asymmetry between the representational and grammatical status of stems and affixes is argued for. In Aronoff’s model, as in other “lexeme” or word-based morphologies (including so-called “word and paradigm” models – see, e.g., Blevins 2016), the basic unit of linguistic analysis is the lexeme – the stem to which inflectional affixes may be added. Derivational affixes, on the other hand, are associated with rules that form lexemes from lexemes; once formed, a lexeme enters the list of available stems, where morphologically complex and morphologically simple stems hold equivalent statuses. The representations of derivational and inflectional affixes fall into different sets of principles and generalizations, and both contrast with the representation of lexemes, which are the only computational “units” in a sense relevant here.

A similar distinction between affixes and stems^1^ is longstanding in the psycholinguistics literature, although not always explicitly so. Taft & Forster’s (1975; 1976) extremely influential Affix-Stripping Model doesn’t actually discuss the representational or further processing status of the stripped away affixes, but by implication, they are peripheral, and play no role in the organization of the mental lexicon. Over the subsequent decades, this distinction between central, richly represented stems (lemmas) and more peripheral, more form-based affixes has assumed a relatively default/uncontroversial status for researchers who have subscribed to the view that morphologically complex words are decomposed during parsing, and that there is such a thing as a morpheme.^2^ The question of just how affixes are stored in long-term memory, and how access to these long-term memories occurs in language processing, has rarely received significant discussion within the psycholinguistics literature. A notable recent exception is Crepaldi et al. (2016) who, while finding significant facilitation in a masked priming study from a pseudoword prime comprising an existing stem and suffix (e.g., *sheeter*) to a real word target ending with the same suffix (e.g., *teacher*), equivalent to the well-established masked-stem-priming effect, also find that this effect is position sensitive for affixes but not for stems (*ersheet* does not prime *teacher*). They argue that these results support a distinction between form-independent lemma-level representations for stems, and lower-level form-dependent “morpho-orthographic” representations of affixes.

In contrast, in a literature associated with Distributed Morphology (DM; Halle & Marantz 1993) the phonological and/or orthographic forms of both stems and affixes are the grammatical realization of terminal nodes in syntactic representations. As such, all stems and affixes are represented as “abstract” units, manipulated by the syntax, and interpreted in form and in meaning at the interfaces of the syntax with the phonology, on the one hand, and the semantic system, on the other. There is no direct theoretical status to the stem vs. affix distinction in DM; however, what we are calling stems in this paper, and what correspond most directly to lexemes in other theories, usually consist in a DM analysis of a root morpheme and an affix (often phonologically null) that carries a syntactic category feature and thus determines the syntactic category of the combination of root and affix. Similarly, the distinction between inflection and derivational morphology plays no direct theoretical role within DM; however, affixes differ along several dimensions within the theory, which can then account for the paradigmatic behavior of much of what is called inflection in the literature (see Marantz 2016 for discussion).

When we ask, then, whether affixes are linguistic units on par with stems, we are asking whether affixes correspond to abstract combinatorial items whose existence might be diagnosed via identity under repetition. Linguistic theories that are “lexeme based” prioritize word-like units and deny morpheme status to affixes on par with lexemes, while “affix-stripping” psycholinguistic models distinguish between central, lemma representations for stems, and peripheral, form-based representations for affixes. By contrast morpheme-based theories in linguistics and psycho/neuro-linguistics claim parallel morpheme status for both stems and affixes. Priming offers an experimental paradigm to decide between these competing theories to the extent that priming can be attributed to the repetition of morphemes and not to the similarity between prime and target along other dimensions that are known to modulate reaction time on the target in the context of the prime.

In this paper, we compare long-lag and immediate priming in an effort to establish that long-lag priming, unlike immediate priming, is driven by the identity between prime and target, rather than by their similarity. On the other hand, form and semantic similarity modulate priming when the target immediately follows the prime. For immediate priming, theories that posit spreading activation from the prime to related lexical entries via shared features seem to capture many of the properties of priming experiments in the literature. In contrast, the properties of long-lag priming in our experiments place long-lag effects within the theory of recollection memory (in which a specific representation, including key contextual information, is reactivated) and outside accounts based on spreading activation (due to similarity relations between representations). For example, the number of intervening words between prime and target does not modulate long-lag priming. Our experiments thus place the theory of morphemes within the psychological literature on the memory for and recollection of other mental objects (see Yonelinas 2002 for a comprehensive overview of the distinction between recognition based on recollection of details, and recognition based on stimulus similarity).

One set of results that has been used to argue for representations in which the stem is itself a unit is long-distance or long-lag morphological (stem) priming, whereby a target that shares a stem with and is therefore morphologically related to a prime presented many words previously yields a speeded reaction time. Primes similar to their targets in form or meaning do not yield anywhere near the magnitude of priming for long-lag priming as primes that contain the same stem as the target. Moreover, as we will explain, our understanding of long-lag priming would seem to require the notion of repetition of a unit, rather than, say, spreading activation over features of a unit or stored representation of a token of a related unit. Masked priming for affixes has also been used to argue for affixes as representational units (Duñabeitia, Perea & Carreiras 2008; Crepaldi, Hemsworth, Davis & Rastle 2016), as discussed above.

Since at least Scarborough, Cortese, and Scarborough (1977), it has been known that participants are generally faster and more accurate in making lexical decisions to test words which they previously encountered a few minutes or even hours previously in a study list. Long-lag repetition priming has been shown to be abstract (robust to form variation) in a series of papers by Bowers and colleagues (Bowers 1996; Bowers 2000; Bowers and Kouider 2003). Long-lag repetition priming effects have also been obtained for pseudowords (e.g., prime: *kers*, target: *kers;* Bowers 1994). More recently, Almeida and Poeppel (2013) compared long-lag priming for words and pseudowords over a lag varying between 9 and 25 items and used MEG to record brain activity while participants performed a lexical decision task. Almeida and Poeppel found significant reaction time (RT) priming for all repeated items, but also an interaction, such that the real word priming effect was larger than for pseudowords. In the neural data, they found that an early response component understood to be related to orthographic processing was modulated by word repetition, but not pseudoword repetition, while a later response component understood to be related to lexical or post-lexical processing was significantly modulated by repetition for both kinds of stimuli.

### 1.1 Long-lag morphological priming: stems

The long-lag priming paradigm was first extended to repeating morphemes (rather than whole words and pseudowords) by Stanners et al. (1979), who compared whole word repetition priming with priming between a prime verb inflected with -*s*, -*ing*, or -*ed*, and an uninflected target. Using visual presentation, and a lag between prime and target varying between 6–15 trials, Stanners et al. found a large repetition priming effect, with no difference between whole word and stem repetition, and no difference between the different inflections.

Marslen-Wilson and Tyler (1997) used an auditory version of the long-lag paradigm to compare morphological priming to semantic (e.g., prime: *doctor*, target: *nurse*) and phonological (e.g., prime: *gravy*, target: *grave*) priming. Their morphological primes were either regular or irregular past tense verbs, with uninflected/present tense targets, and primes preceded their targets by 12 items. They found robust priming effects for both the regular and irregular inflection conditions (no difference between them), and the semantic condition, but no priming for the phonological condition. Feldman (2000) compared uninflected verb targets (e.g., *vow*) preceded by regular past tense inflected primes (*vowed*), semantic primes (*pledge*), or orthographic primes (*vowel*) with visual stimulus presentation and a prime-target lag of 10 and found significant priming only for the morphologically related condition.

Feldman and Moskovljevic (1987) investigated long-lag morphological priming in Serbo-Croatian,^3^ which can be written using either a Roman or Cyrillic alphabet, and manipulated whether the prime and target were in the same or different orthography. They found robust long-lag morphological priming for differently inflected case forms of nouns (e.g., prime: *dinar*, target: *dinaru*) with an average lag of 10 items, and no effect of the orthographic system manipulation, which supports the view that long-lag priming effects are due to abstract, form-independent representations.

Kouider and Dupoux (2009) conducted a long-lag morphological priming experiment in French. Their morphologically related condition consisted of pairs of French feminine and masculine adjectives (e.g., prime: *gamine*, target: *gamin*), and they also included phonological, semantic, and full repetition conditions, as well as a pseudo-morphology condition in which nonword stems were inflected with feminine and masculine affixes (e.g., prime: *janoise*, target: *janois*). In three separate auditory lexical decision experiments, they varied the lag between prime and target from “short” (12–24 intervening items, mean of 18), to “medium” (48–96 intervening items, mean of 72), to “long” (96–192 intervening items, mean of 144). The full repetition and morphology conditions were associated with significant priming effects across all three experiments, with no significant priming for the other conditions in any experiment. They found that the magnitude of the morphological priming was consistent across all three experiments, while the magnitude of the full repetition priming was greater than for the morphological priming at the short and medium lags but was the same at the longest lag.

Wilder et al. (2018) found very similar results to Kouider and Dupoux (2009) at much shorter lags. They compared morphological priming (prime: *frogs*, target: *frog;* prime: *frog*, target: *frogs*), repetition priming (prime: *frog*, target: *frog*; prime: *frogs*, target: *frogs*), and phonological priming (prime: *grape*, target: *gray*; prime: *gray*, target: *grape*), with lags of zero, one, and five items between the prime and target in an auditory lexical decision experiment. In the zero and one lag conditions, they obtained significant priming effects for both morphological priming and repetition priming and found that repetition priming effects were significantly larger than morphological priming. Phonological priming was found only in the zero lag condition. At the longest lag (five), the difference between morphological and repetition priming disappeared, with equally robust priming effects for both morphological and repetition priming. As in Kouider and Dupoux (2009)’s experiments, the magnitude of the morphological priming effect remained similar over the different longer lag conditions, while the repetition priming effect decreased over increased distances.

Across the set of experiments, the consistent finding is that long-lag morphological priming for stems is found over distances of as many as 192 intervening trials (Kouider & Dupoux 2009), with no change in magnitude of priming, and that there is no difference between stems with regular and irregular inflection (Marslen-Wilson & Tyler 1997). Priming between phonologically or semantically related words does not generally occur over these long lags. Interestingly all these experiments involve inflectional morphology rather than derivational. Whether morphological priming looks like identity priming depends on the experiment. Stanners et al. (1979) found no differences, but Kouider and Dupoux (2009) and Wilder et al. (2018) found that identity priming decreased slightly over the longest distances, while morphological priming remained consistent.

### 1.2 Long-lag morphological priming: affixes

We turn, then, to long-lag morphological (affix) priming to ask whether we can find evidence that derivational suffixes in English are units of representation in the same sense that words and stems seem to be. Although they share a suffix that turns adjectives into verbs, the overlap in form and meaning between pairs like *diversify* and *intensify* is not particularly striking, especially compared to the overlap among *diverse*, *diversity*, *diverseness*, etc. that involves similarities among derivatives of the same stem. Unless repetition of the unit -*ify* as a unit is particularly significant for processing, we would not expect a long-lag affix priming effect in lexical decision. Contemporary linguistic theory, however, insists that -*ify* is a combinatorial unit in the same sense as *diverse.*

Note that pinning down a consistent semantics for many such derivational morphemes in English is often not possible. -*ize* can be used with a meaning of ‘make or become’ (e.g., *fossilize*), ‘treat in a specific way/combine with’ (e.g., *carbonize*), and ‘follow or subject to a practice’ (e.g., *theorize*, *hospitalize*). What is critical to the argument that -*ize* is nonetheless a grammatical combinatorial unit is that it has a consistent phonology and syntax (-*ize* is pronounced consistently with the same phonemic sequence and does not shift stress on the stems to which it attaches; -*ize* suffixation creates transitive verbs with theme/patient internal arguments). In a long-lag paradigm, where effects of semantic similarity are not expected to persist (Feldman 2000; Kouider & Dupoux 2009), only the grammatical identity between prime and target is expected to matter, not the semantic or conceptual similarity.

Unless there are special processing considerations relevant to memory and recollection of affixes that do not apply to stems, we as linguists expect to find long-lag priming for affixes of the same sort as that found for repetition of stems. VanWagenen (2014) tested this prediction in a long-lag morphological (affix) priming experiment. The experiment builds on immediate priming experiments such as Marslen-Wilson, Ford, Older, and Zhou (1996) who used a cross-modal (auditory prime, visual target) paradigm and both prefixed and suffixed stimuli and found a priming effect for shared-affix pairs (prime: *happiness*, target: *darkness;* prime: *rearrange*, target: *rethink*), and no effect for pseudo-affixed pairs (prime: *happiness,* target: *harness;* prime: *rearrange,* target: *recent*). Marslen-Wilson et al. (1996)’s effect was only significant for affixes judged to be productive, but there was still a trend to priming even for the affixes judged to be unproductive.

VanWagenen (2014) compared morphological (prime: *humanism*, target: *heroism*), form (prime: *heresy*, target: *heroism*), and semantic priming conditions (prime: *valor*, target: *heroism*), and used 40 different derivational affixes to avoid any repetition outside the prime-target pair. VanWagenen used rigorous criteria for selecting materials: only derived words with transparent relations with their bare/stem form were included (e.g., *virtual* was not included because it is not transparently related to *virtue*), and only pairs of words whose affixes make the same contribution to meaning were included (e.g., as discussed above, the -*ize* in *idolize* and the -*ize* in *philosophize* contribute different meanings, so such pairs were not included). The prime to target distance ranged from 5 to 33 items (with an average of 20). VanWagenen (2014) found a significant, 20 ms morphological priming effect, and no priming for the semantic or form prime conditions, exactly as predicted if affixes are stored representational units.

To avoid confusion, from this point forward in the paper we will refer to morphological (affix) priming simply as affix priming, while continuing to refer to morphological (stem) priming as morphological priming because this has been customary in the literature.

It is important to note a crucial difference between VanWagenen’s long-lag affix priming paradigm and the long-lag “morphological” (stem) priming experiments reviewed above. In previous long-lag morphological priming studies, although each stem (e.g., *talk*) would be encountered at most twice (e.g., in prime *talked*, as well as in the target *talk*), the affixes would be repeated many times. Thus, recovering the stems (which were the experimental objects of interest) involved repeatedly stripping the same affixes from different inflected items. In VanWagenen (2014), as in previous long-lag morphological priming experiments, the experimental objects of interest (now the suffixes, rather than the stems) appear at most twice, but the stems that need to be stripped to recover the affixes occur only once rather than being repeated many times. If we think of each affix as involving a morphological “rule” in some sense, the same rule or rules are used over and over in the stem priming experiments, while in the affix priming experiment, each rule is used at most twice. This difference in design biased VanWagenen’s experiment against finding an affix priming effect, because we expect that repeated application should facilitate the morphological rule. In our elaborations of VanWagenen’s pioneering experiment, we asked whether we would see a stem priming effect for derived items if, as with VanWagenen’s affix priming experiment, subjects only saw the same affix – and used the same morphological rule – once.

We should not be content, however, merely to demonstrate an effect of affix repetition in long-lag lexical decision. There are hallmarks of long-lag priming indicative of the processing of units that we should observe for affix priming, if we’re seeing true repetition priming. First, although “spreading activation” is one plausible mechanism for short-lag priming, it is not plausible for long-lag priming, for reasons explained in the literature (Kouider & Dupoux 2009; Dannenbring & Briand 1982): residual activation of features should show decay over time, whereas there is no apparent effect of distance for the reactivation of a representation involved in recollection memory. We should therefore not see any prime-target distance effect on affix priming if it is in fact repetition priming, involving recollection memory of a specific representation.

However, for recollection memory, research has found an interaction between repetition priming and the frequency of the prime/target: the lower the frequency of the item, the greater the priming effect (Norris 1984). The logic behind this frequency effect is intuitive – in recollecting that you’ve encountered an item before, the noticeability of the first encounter makes the item more memorable, and the lower the frequency of a word, the more noticeable it is. For affix priming, the relevant numerical index of noticeability is not obvious. It could be that the frequency effect for repeated words holds directly for repeated affixes, such that affix frequency would be predicted to interact with repetition in modulating RT for lexical decision. However, since we know that a different measure of predictability, the transition probability from stem to suffix, modulates brain activity at early stages of lexical access in reading (Gwilliams & Marantz 2016), we might also hypothesize that the lower the transition probability, the more noticeable the affix, and thus the more memorable. Thus, lower transition probability might correlate with a greater priming effect in long-lag affix priming.

In a series of eight experiments, we replicated VanWagenen (2014)’s long-lag affix priming effect and compared it to five other types of priming at long and short lags, in order to further explore what exactly it means to activate and reactivate a morpheme (especially an affix), and what memory mechanisms are involved. In Experiment 1, we conducted a replication of VanWagenen (2014) and found, as she did, evidence for affix but not semantic or form priming at long lags. With target *heroism*, we found long-lag priming from *humanism* (affix prime) but not from *valor* (semantic prime) or *heresy* (form prime).

In Experiment 2, we replaced our affix priming condition with an identity priming condition (in which prime and target were identical), for comparison with the partial identity relationship occurring in affix priming. Having established robust long-lag affix and identity priming effects in the absence of semantic and form prime effects, we then decided to use the same paradigm to examine traditional morphological priming effects, in which the stem is the repeated unit. The semantic and form prime conditions were always included for comparison. In Experiment 3, we used the stem as the prime and kept the derived form as the target, and in Experiment 4 we reversed this. As described above, morphological (stem) priming effects have been found repeatedly in the literature, but only in paradigms using frequently repeated inflectional affixes. We were surprised to find that in our paradigm, with derivational affixes and with no repetition of units beyond the specific prime-target repetition, morphological (stem) priming effects were weak or not present.

Our studies do not directly compare affix, identity, and stem priming within the same experiment, although this would be useful in future work. We also did not consider stem priming in which prime and target were both derived forms with different affixes, although again, this would be a useful datapoint.

In Experiments 5–8, we repeated Experiments 1–4 with short-lag (immediate) rather than long-lag priming, a design for which semantic and form priming effects are strongly expected to occur. This was done to ensure that any lack of semantic and form priming effects at long lags was due to the lag and not because the primes were not closely related enough. We found robust affix, identity, stem-to-derived, derived-to-stem, form, and semantic priming effects in this immediate priming design.

## 2 Methods

### 2.1 Materials

#### 2.1.1 Experiment 1

Experiment 1 was intended to replicate and extend the findings of VanWagenen (2014). Therefore, we used identical targets and affix, semantic, and form primes. Table 1 summarizes relevant stimulus properties for each condition; a complete list of stimuli and individual item characteristics are available in the supplementary materials for this paper on the Open Science Framework (OSF). The condition we label “affix” priming is VanWagenen’s “morphological” priming condition. Per VanWagenen, the target condition was comprised of 40 bimorphemic words (e.g., *heroism*) with 40 unique suffixes, so that no suffix could be primed by any other target.

The affix primes (e.g., *humanism*) contained the same suffixes as the targets but with different stems and were matched with targets on the basis of phonological stem changes. VanWagenen (2014) reports that four linguists independently evaluated the targets and affix primes to ensure that the relationship between stem and derived form was semantically transparent. Additionally, two linguists considered the semantic contributions of the affixes and ensured that the affix was making the same semantic contribution to both the derived target and derived affix prime within each pair.

For form primes (e.g., *heresy*), VanWagenen ensured that the number of overlapping character/phonological segments between form prime and target was the same or greater than the number of overlapping segments between affix prime and target. However, in the affix priming condition, the overlap with the target was always due to the shared suffix and therefore occurred at the end of the word. In the form priming condition, the location of overlap was not restricted. An orthographic Levenshtein distance measure was used to generate form prime candidates that involved at most four character deletions, insertions, or substitutions when compared to the target. For these orthographically constrained words, phonemic transcriptions were then compared by hand so that form primes could be selected on the basis of phonological Levenshtein distance. The final set of affix primes and targets had an average of 3.75 overlapping characters and 3.20 overlapping phonemes; the final set of form primes and targets had an average of 5.23 overlapping characters and 4 overlapping phonemes. The overlap did not have to be contiguous.

Finally, VanWagenen used Latent Semantic Analysis (LSA) scores (Landauer, Foltz & Laham 1998) to ensure that semantic primes (e.g., *valor*) were as semantically related as possible to the targets, and substantially more semantically related to the targets than affix primes were. Synonyms and associates from WordNet (Fellbaum 1998) were used as candidates, and the word with the highest LSA relatedness score (while also closely matched for frequency and number of syllables) was selected. For semantic primes and targets, the mean LSA score was 0.19, while for affix primes and targets, the mean LSA score was 0.08.

VanWagenen (2014) created four lists for stimulus presentation. The task in this experiment was continuous lexical decision, so the trial structure did not make prime-target pairings obvious to participants. Prime and target occurred at some distance from each other, interspersed with other primes and targets as well as nonwords. Targets were divided into four groups of 10, and within each list, each of the four groups was assigned a different prime condition (affix, form, semantic, and no prime). The prime condition assigned to each target group rotated across the four lists, such that each target was presented only once in each list but the assignment of targets to conditions was counter-balanced. In the affix, form, and semantic prime conditions, the target followed a matched prime at some distance in the presentation list. In the no-prime condition, there was no specific preceding item included as the prime. This resulted in a total of 70 real words per list (40 targets, 10 affix primes, 10 form primes, 10 semantic primes). Among these real words, 30 suffixes appeared once, and 10 suffixes appeared twice (when their target was in the affix prime condition and the prime therefore shared the same suffix).

A single set of 70 pronounceable nonwords was created to mimic the parameters of the real word list and make the lexical decision task as engaging as possible. The nonwords used in VanWagenen (2014) were no longer available, so a new set of nonwords conforming to the standards described by VanWagenen (2014) was created using the freely available software Wuggy (Keuleers & Brysbaert 2010). Nonwords, with an additional 40 (real) derivational suffixes distinct from the 40 suffixes used in the targets, were matched with real words for length and number of syllables and matched for transition frequencies within the words to the greatest extent possible. 10 of these suffixes were repeated, so that the distribution of suffixes would be the same as that of the real words. A complete list of nonwords used in the current experiment is included in the supplementary materials on OSF.

In each of the four lists VanWagenen (2014) divided the 140 items (70 words and 70 nonwords) into four blocks of roughly 35 items each, and pseudo-randomized the order of items within each block. This was done to ensure that within each block the primes not only preceded the targets but were also separated by the necessary distance. In that experiment, the minimum distance was five items, the maximum distance was 33 items, and mean distance was 20 items. The order of items within each block was fixed in each list, but the order of presentation of blocks was random.

In our experiment we followed the same basic principles but introduced several variations in presentation to minimize the possibility of list and order effects. First, we started with a random group division (designating which targets would appear together in each condition) different from the division used in the original experiment, and subsequently a random block division (designating which targets would appear together in each chronological block) also different from the division used in the original experiment. These group and block designations were kept the same across all presentations of the current experiment. The Latin Square design then assigned each participant to a “list” (determining which group of targets would appear in each condition), such that the condition of each target would vary based on the list assignment, but the targets appearing in each presentational block would not vary. Like VanWagenen, we randomized the order of blocks with each run of the experiment.

Second, we randomized the order of presentation within each block with each run of the experiment, restricting the randomization only to ensure that primes preceded targets and prime-target distance was randomly varied. This had the effect that prime-target distance varied continuously from 1 to 34 both between and within items. In VanWagenen (2014), because pseudo-randomized orders within blocks were frozen within lists, each target always appeared at the same distance from its prime in any given condition.

#### 2.1.2 Experiments 2–8

In addition to Experiment 1, which was intended to replicate VanWagenen (2014), we conducted seven follow-up experiments to allow for comparison with different types of priming. Each follow-up used exactly the same stimuli and presentation details except for a single key change.

In Experiment 2 we considered the effect of identity priming at long lags. To do this, we replaced the affix priming condition with an identity priming condition, in which the primes were identical to the targets (i.e., *heroism* appeared once as prime and once as target). The semantic, form, and no-prime conditions stayed the same.

In Experiment 3 we considered the effect of stem priming at long lags, replacing the affix/identity priming condition with a stem priming condition, in which the primes were the stems of the derived targets, with no suffix (e.g., prime: *hero*, target: *heroism*). The semantic, form, and no-prime conditions were again unchanged. It was not possible to use derived forms with the same stems as primes because we would have needed 40 additional unique affixes, most of which would then have to be quite low frequency.

In Experiment 4 we reversed the stem priming experiment, using the stems as targets and the derived forms as primes. To do this, we replaced the affix/identity/stem priming condition with the derived target words and used the stems as targets instead (e.g., prime: *heroism,* target: *hero*). The semantic and form primes were the same, but their targets were necessarily the stems rather than the derived forms, as in the previous studies (e.g., primes: *valor* and *heresy*, target: *hero*).

In Experiments 5–8, we repeated Experiments 1–4 but removed the lag between prime and target. In these designs, the prime and target always occurred consecutively (never with other primes, targets, or nonwords intervening), following conventions in the literature for lexical decision experiments examining overt, immediate repetition priming.

### 2.2 Procedure

The protocol for all studies was approved by the Institutional Review Board at New York University, ID #15–10698. A continuous lexical decision task^4^ was presented using the freely available experiment presentation software Ibex^5^ and distributed via Amazon Mechanical Turk. Each study was posted on Amazon Mechanical Turk’s online marketplace as a *Human Intelligence Task* (HIT) between July and November 2015. We included a brief description of the task and warned that only participants working from keyboards (and not touch screens) would be able to complete the task. $0.50 was offered for completing the study, which was expected to take 5–10 minutes. Several restrictions were placed on worker eligibility: only U.S. IP addresses were allowed, the worker’s HIT approval rate for all previously completed HITs had to be greater than or equal to 97%, and the total number of HITs approved for the worker had to be greater than or equal to 5000. We also ensured that workers could not participate in more than one of our experiments.

After clicking on the posting, workers were linked to an IRB-approved consent form and were instructed not to proceed with the experiment unless they had read and understood the consent form and were willing to participate. They were also informed that after completing the experiment they would be asked a series of six demographic questions, but that all information collected would be strictly anonymous. Those who opted to continue were then presented with a link to the experiment on the Ibex server.

The experiment was presented in white text on a black screen. Participants were instructed that they would see a string of letters across their computer screen, and then were to decide whether or not the string was a real word of English by pressing *K* on their keyboards for real word and *S* for nonword, responding as quickly and as accurately as possible.

They then completed a practice round of 30 trials (with no overlap between practice items and experimental items), before being presented with the instructions for a second time and then pressing any button when they were ready to begin. Each trial consisted of a fixation cross lasting for 500 ms, and then the presentation of the word or nonword, which remained on the screen until a response was logged. The four blocks were presented without breaks and took 5–10 minutes in total to complete. At the end of the experiment, a completion code appeared on the screen and participants were instructed to copy this code into the window of their Mechanical Turk HIT in order to receive credit for having completed the task. They were then asked for information about age, gender, and language background. The Ibex completion code entered in the HIT thus served to connect Ibex results to the demographic information provided by each participant.

A known problem with the interface between Ibex and Amazon Mechanical Turk (during our data collection, in 2015) was that the Latin Square mechanism in Ibex relies on a counter that increments with each completion of the experiment. Because the nature of the Mechanical Turk workplace is that many workers click on and commence a HIT in the first few minutes after it is posted, this can result in many workers completing the same list version before the counter is incremented. We therefore slightly altered the experiment design in Ibex so that the counter was incremented as soon as the experiment link was clicked by a worker. This did not, however, completely resolve the issue, resulting in our list assignments being not completely balanced. The number of observations for each item is available in the supplementary materials for this paper on OSF, and the range for the number of observations included in the analysis for each target item is reported in Table 2.

### 2.3 Participants

As described above, our participants were workers on Amazon Mechanical Turk. VanWagenen (2014) had a sample size of 66, and as we were attempting to replicate this experiment, we intended to match the sample size. Because we were unsure about the quality of priming data collected online and did not know what percentage of collected datasets would be useable, the HIT we posted for Experiment 1 was intended to allow for 100 unique completions. Technical problems with the Ibex server and with the interface between Ibex and Amazon Mechanical Turk resulted in 67 logged datasets, 57 of which were useable. When Experiment 1 resulted in an affix priming effect size (−0.35) very similar to that found in VanWagenen (2014) (−0.28), we decided to continue using this sample size in our follow-ups (see Table 2). Experiments 3 and 7 are exceptions because experimental error resulted in multiple postings of the HIT on Amazon Mechanical Turk.

We did not conduct power analyses before running these experiments, but a post-hoc power analysis using the effect size for long-lag affix priming found by VanWagenen (2014) indicates that our Experiment 1 had 58% power to detect that effect size. Semantic and form priming were not expected to occur. We expected long-lag identity and stem priming to have similar if not larger effect sizes than affix priming, and so we are similarly powered in Experiments 2 and 4 with their comparable samples. Experiment 3, due to the accidental multiple postings and resulting sample size of 108, had 85% power to detect an effect of the size reported by VanWagenen (2014). Because the short-lag studies do not have an effect size to compare with in VanWagenen (2014), we cannot speak to our statistical power in these cases. Using an effect size from a study with different stimuli would be possible but not directly comparable. If anything, we expect all of the short-lag effects to be bigger, and so with the same sample sizes we are more adequately powered in those cases.

### 2.4 Data Processing

Data pre-processing was conducted using Eelbrain (Brodbeck & Das 2019) in Python. The details in this section apply to all eight experiments. Based on the demographic information provided at the end of the HIT, participants who indicated that English was not their native language were excluded from analysis. Next, participants who appeared to have completed the experiment twice, for unknown reasons, were removed. Finally, for each participant, for each target condition (affix/identity/stem/derived prime, form prime, semantic prime, or no prime) we calculated the mean reaction time (RT) and standard deviation. We then removed any participant whose mean RT in any one condition was greater than 2.5 standard deviations from the mean for that condition across participants.

After excluding targets with incorrect responses and those whose primes had incorrect responses, any remaining individual reaction times that were further than the cut-off value of 2.5 standard deviations from the subject’s mean RT for accurate word responses were replaced with that cut-off value. This approach to outlier replacement is identical to the approach used by VanWagenen (2014). Table 2 reports, for each experiment, the number of datasets logged and analyzed, as well as accuracy for primes and targets (collapsed over condition) and the percentage of individual reaction times excluded. In an exploratory alternative analysis, we used a more conservative protocol that simply excluded reaction times faster than 150 ms or slower than 1500 ms.

Note that until this point we have referred to a “list” as the full set of items presented to a given participant. However, from this point forward, in order to align our statistical models with those of VanWagenen (2014), we will refer to “list” as the set of 10 targets which always appear in the same priming condition, and “group” as the full set of items presented to a given subject.

### 2.5 Analysis

All statistical analysis was conducted in R (R Core Team 2018). In alignment with VanWagenen (2014), we first computed simple by-participant means in each priming condition in each experiment. For comparison between the no-prime condition and each of the priming conditions, we computed effect size^6^ (Cohen’s *d*, with non-pooled standard deviation and a non-central *t* distribution) and a Bayes factor^7^ indicating the relative evidence for a difference between the two conditions over a null effect. Following VanWagenen (2014), for these comparisons we excluded trials on which the distance between prime and target was less than 5, so that they actually reflect long-lag priming.

We then conducted linear mixed effects regression (LMER) analyses (on single trials rather than participant means), with separate models for long-lag and short-lag data. We did not transform reaction times, following the recommendation of Liceralde and Gordon (2018). In addition to prime type, we included predictors in these models (as main effects) that were not related to our hypotheses about priming but are known in general to influence reaction time in lexical decision. These were: whether or not the response to the previous item was accurate, length of target affix, length of target, whether or not the previous item was a nonword, order in experiment, (scaled) RT to previous item, and (scaled) RT to prime. Accuracy and nonword status of the previous item were only included for long-lag conditions, since for short-lag conditions the previous item is the prime. We also included the group, list, and experiment in which the target appeared. All factors were treatment-coded. Unless otherwise noted, the reference level for the prime type factor was the no-prime condition. The reference levels for whether or not the previous item was a nonword and whether or not the response to the previous item was accurate were both *no*. The reference levels for the list and group factors were *List 1* and *Group 1*, and for the experiment factor was the experiment that included identity priming.

Variables related to our hypotheses such that their effects might differ by condition were evaluated for interactions with prime type. The first was prime distance (for the long-lag data only). Recollection priming does not predict an effect of prime distance but spreading activation accounts do. The rest are related to the expectation from recollection priming accounts that lower frequency should lead to increased priming (meaning faster reaction times): (log) frequency of target affix, (log) surface frequency of target, (log) transition probability of target suffix given target stem, (log) transition probability of prime suffix given prime stem.

All frequency-related variables were extracted or calculated from CELEX (Baayen, Piepenbrock & Gulikers 1995). Transition probability of the suffix given the stem was calculated as surface frequency divided by lemma frequency. We defined lemma frequency as the lemma frequency for the target wordform as identified by CELEX, plus the CELEX lemma frequency of the stem. For the surface frequency and affix frequency variables, we dealt with missing values in CELEX by adding 1 to all frequencies, so that missing items had a frequency of 1 rather than 0. However, we used raw lemma and surface frequencies to compute transition probability of the suffix. Therefore, items whose lemma or surface frequencies were missing from CELEX were excluded from the analysis that included transition probability of the suffix. In an exploratory alternative analysis, we instead used MorphoLex frequencies (Sánchez-Gutiérrez, Mailhot, Deacon & Wilson 2018).

For all models, we included random intercepts for subject and word, after failure to converge of an initial model that also contained random slopes for prime type by subject and word. We used likelihood ratio tests to determine whether fixed effects contributed significantly to the fit of a model,^8^ following Barr et al. (2013). We removed non-significant interactions and refit our models to improve the interpretability of main effects. In the Results section, we report the outcomes of likelihood ratio tests, with model estimates for continuous variables and factors with only two levels. Complete model outputs are provided in the supplementary materials on OSF. When the effect of the prime type factor (or an interaction with it) was significant, we conducted follow-up pairwise tests^9^ between the estimated marginal means (see Searle, Speed & Milliken 1980) for each priming condition and the no-prime baseline, averaged over the levels of the other factors in the model. We adjusted for multiple comparisons within each set of contrasts using a multivariate *t* distribution. We did not compute estimated marginal means for the remaining multi-level factors (group, list, experiment) as they were not relevant to our critical questions.

## 3 Results

We first present reaction time and accuracy data by experiment in Tables 3 and 4. In our Experiment 1, effect sizes in each priming condition are comparable to those observed by VanWagenen (2014). Reaction times in all conditions are somewhat slower than was observed in VanWagenen (2014). This is likely attributable to the increased variability (in participant characteristics, hardware used to make responses, etc.) associated with online data collection.

### 3.1 Experiments 1–3

**Figure 1:**
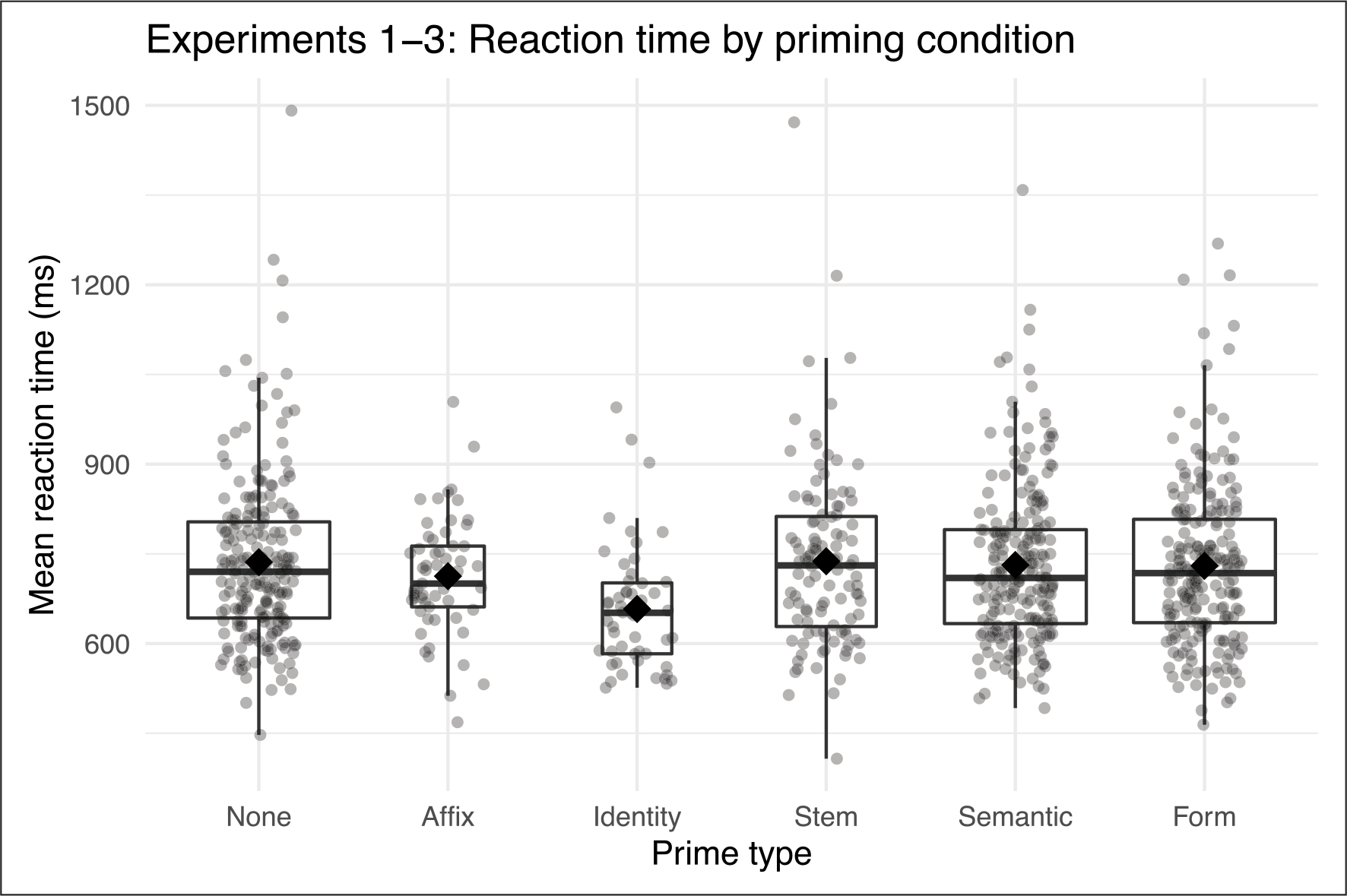
Mean reaction times (ms) for Experiments 1–3 (long-lag priming). Upper and lower hinges of boxplots indicate 75% and 25% quartiles with line at median. Whiskers extend from hinge to largest and smallest values within 1.5* the inter-quartile range. Diamonds indicate group mean and grey dots indicate participant means. Boxplot width reflects number of observations.

For our regression analyses we pooled the data from Experiments 1 through 3, since these three experiments involved the same set of targets and the same long-lag paradigm. The conditions included as levels in our prime type factor were therefore: no prime, affix, semantic, form, identity, stem. Figure 1 shows mean reaction times for these conditions, collapsed across experiments.

We first evaluated a model with all variables relevant to all of the data. Interactions between prime condition and surface frequency, affix frequency, transition probability of the suffix, and affix length did not significantly improve model fit, so we refit the model with main effects only for these variables. We then conducted follow-up pairwise comparisons between the estimated marginal means for each of the priming conditions and the no-prime baseline, finding significant effects of affix priming and identity priming but not stem, semantic, or form priming (see Table 5).

Experiments 1–3 therefore replicate VanWagenen (2014) in showing long-lag affix priming, as well as in not showing long-lag semantic or form priming. Identity priming at long lags is strongly exhibited, with a larger estimate than affix priming. Finally, we do not see priming from the stem. Because we were specifically concerned with whether affix priming effects exceed any potential effects of semantic or form similarity, we also conducted follow-up pairwise comparisons between the estimated marginal mean for the affix priming condition and those for the semantic and form priming conditions. Reaction time was significantly faster in the affix priming condition than in the semantic priming (contrast estimate = −24.8, *SE* = 11.0, *z* ratio = −2.251, *p* = .039) and the form priming condition (contrast estimate = −24.9, *SE* = 11.1, *z* ratio = −2.239, *p* = .040). Finally, reaction time in the affix priming condition was not significantly different from reaction time in the stem priming condition (contrast estimate = −15.7, *SE* = 12.8, *z* ratio = −1.223, *p* = 0.22). In Table 6, we report the results of likelihood ratio tests for all fixed effects in the main model, with estimates where applicable.

We also performed a follow-up test on the subset of targets that were primed (excluding the no-prime condition) adding two prime-specific predictors and their interactions with prime type: reaction time to the prime, and distance between prime and target. We determined via likelihood ratio test that model fit was not significantly improved by the interaction between prime type and reaction time to the prime (χ^2^(4) = 9.19, *p* = .057), so we re-fit the model with a main effect for this predictor. We found a significant interaction between prime type and prime distance (χ^2^(4) = 10.05, *p* = .040), and no significant effect of (scaled) reaction time to the prime (χ^2^(1) = 2.31, *p* = .129). Though the identity priming condition exhibited a numerically larger estimated effect of prime distance than the other priming conditions, there were no significant differences in pairwise comparisons. Complete information for these analyses is reported in the supplementary materials on OSF.

We attempted to perform a final follow-up test on the affix-primed targets only, to assess the effect of the transition probability of the suffix for the prime on reaction time to the target. However, this resulted in a model with singular fit, likely due to insufficient data in this single condition.

### 3.2 Experiment 4

We analyzed Experiment 4 in a separate model because for this experiment the targets were stems, while in Experiments 1–3 they were the derived forms. The relationship between prime and target for the semantic and form priming conditions was therefore different from that in Experiments 1–3. This experiment was conducted with derived primes and stem targets because this is a common setup in morphological priming experiments in the literature.

We used the same final model that we had arrived at for Experiments 1–3, removing variables that did not apply (affix frequency, transition probability of the suffix, affix length, experiment). Prime type did not significantly contribute to model fit (see Table 7). Thus, we did not find evidence for long-lag priming of any kind in Experiment 4, with derived primes and stem targets.

### 3.3 Experiments 1–4: alternative analyses

In order to explore the impact of our analysis choices on our pattern of findings, we also investigated whether our main results (for models including all targets) were dependent on our data-cleaning protocol or affected by the use of older CELEX frequencies. First, instead of excluding participants and individual datapoints on the basis of reaction time means and standard deviations, we simply excluded any reaction time faster than 150 ms or slower than 1500 ms. The critical significant effects of affix and identity priming still occurred. Among the previously non-significant effects, we again did not observe semantic, form, or derived priming. However, we did observe a significant effect of stem priming (contrast estimate = 20.52, *SE* = 5.88, *z* ratio = 3.486, *p* = .001). We again did not observe a significant difference between reaction times in the stem and affix priming conditions (contrast estimate = 2.77, *SE* = 8.94, *z* ratio = 0.310, *p* = .757).

Next, while retaining the original data-cleaning protocol, we repeated the regression analyses using frequency values from the MorphoLex database (Sánchez-Gutiérrez, Mailhot, Deacon & Wilson 2018), which uses Hyperspace Analogue to Language frequencies (Burgess & Livesay 1998) as made available by the English Lexicon Project (Balota et al. 2007). Note that lemma (or “root”) frequencies in MorphoLex collapse over syntactic category, unlike those in CELEX. Our pattern of findings for Experiments 1–3 did not change. For Experiment 4, the model using MorphoLex frequencies resulted in singular fit. Full details for all alternative analyses are available in the supplementary materials on OSF.

### 3.4 Experiments 1–4: summary

The results of Experiment 1 replicated VanWagenen (2014)’s finding that there is affix priming but not semantic or form priming at long lags, with a very similar effect size estimate to VanWagenen (2014). In Experiments 2 and 3 we repeated the long-lag priming paradigm, using the same targets, but examined identity priming and stem priming in place of affix priming, each time in comparison with the same semantic and form priming conditions. Experiment 4 used derived primes and stem targets. We found evidence for identity priming but not for priming of stem or derived forms at long lags. However, we did observe a stem priming effect with a more conservative data-cleaning protocol. Across all of the experiments, we did not see any effects of long-lag semantic or form priming. We consider the implications of the other variables we tested, relevant to the recollection memory account of long-lag priming, in the Discussion section.

### 3.5 Experiments 5–7

**Figure 2:**
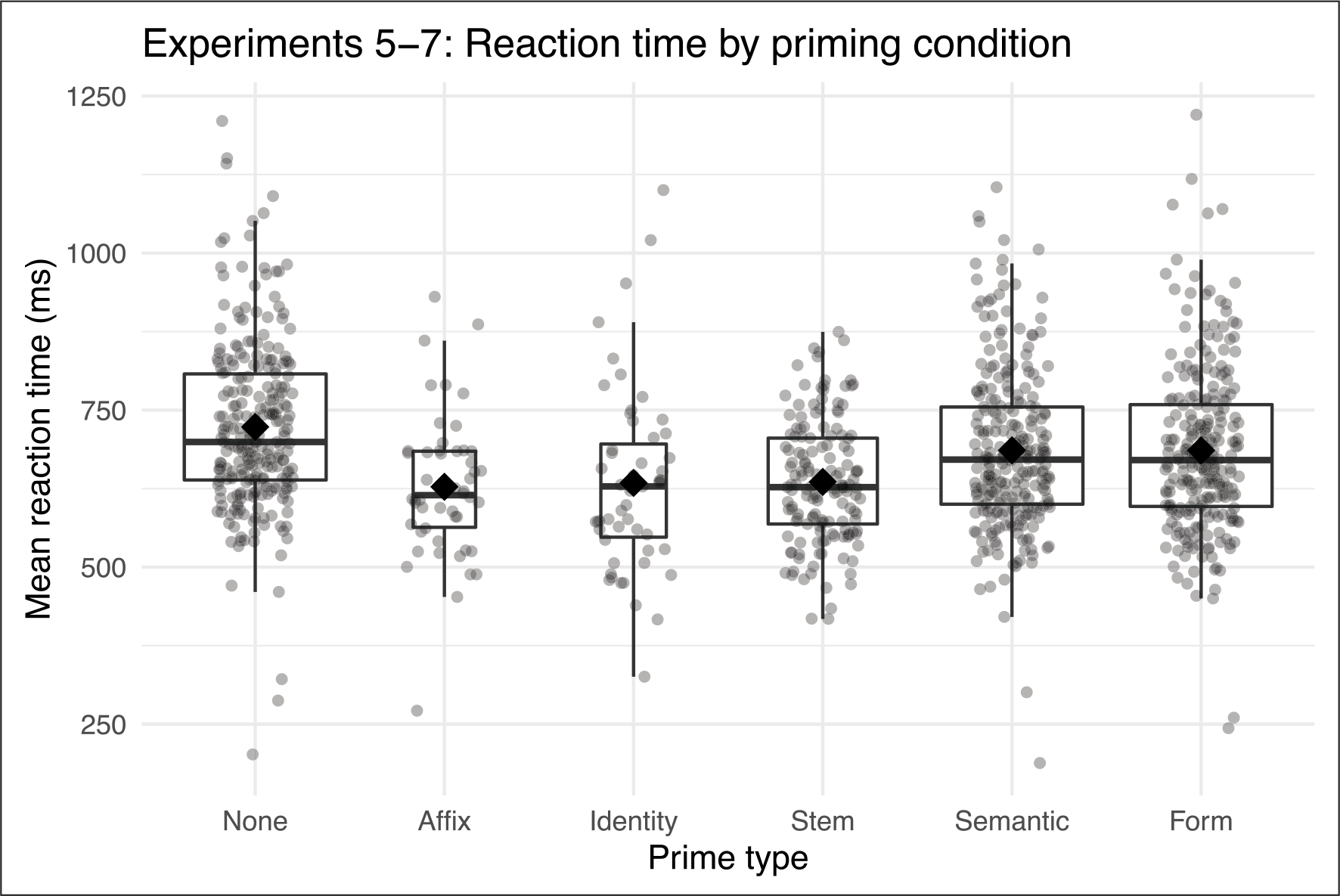
Mean reaction times (ms) for Experiments 5–7 (short-lag priming). Upper and lower hinges of boxplots indicate 75% and 25% quartiles with line at median. Whiskers extend from hinge to largest and smallest values within 1.5* the inter-quartile range. Diamonds indicate group mean and grey dots indicate participant means. Boxplot width reflects number of observations.

We followed the same protocol for analyzing the aggregated short-lag data, considering Experiments 5–7 separately from Experiment 8. We used the same initial fixed effects model, except for removing accuracy and nonword status of the previous word due to the fact that the prime was always the preceding word. Figure 2 shows mean reaction times for each priming condition, collapsed across experiments.

Interactions between prime type and affix frequency, transition probability of the suffix, and affix length did not significantly improve model fit, so we re-fit the model with main effects for these variables. We then conducted follow-up pairwise comparisons between the estimated marginal means for each of the priming conditions and the no-prime baseline, finding significant effects of all five priming types (Table 8). In Table 9, we report the results of likelihood ratio tests for all predictors in the model, with estimates for continuous variables.

Because we observed a significant interaction between prime type and surface frequency, we then computed the estimated marginal trends for the surface frequency effect within each priming condition (Table 10).

There was a facilitatory effect of surface frequency of the target in the no-prime condition. The surface frequency effect was significantly smaller in the affix, identity, and stem priming conditions, but not significantly different from the no-prime condition in the semantic and form conditions.

We did not conduct the same follow-up on primed conditions that we conducted for the long-lag data because prime distance was no longer relevant and reaction time to the prime was encompassed by the reaction time to the previous item in our main model. We did attempt to perform the same follow-up test on the affix priming condition that we attempted for the long-lag paradigm, to assess the effect of the transition probability of the suffix for the prime on reaction time to the target. This again resulted in a model with singular fit.

### 3.6 Experiment 8

As with Experiment 4, we analyzed Experiment 8 in a separate model because the targets were stems rather than derived forms. We used the same final model that we had arrived at for Experiments 5–7, removing variables that no longer applied (affix frequency, transition probability of the suffix, affix length, experiment). The interaction between prime type and surface frequency was again significant. We then conducted follow-up pairwise comparisons between the estimated marginal means for each of the priming conditions and the no-prime baseline (Table 11). We found evidence for short-distance derived, semantic, and form priming. In Table 12, we report the results of likelihood ratio tests for all predictors in the model, with estimates for continuous variables.

Because of the significant interaction between prime type and surface frequency, we also computed the estimated marginal trends for the surface frequency effect within each priming condition (Table 13). There was a facilitatory effect of surface frequency in the no-prime condition, which was significantly larger in the semantic priming conditions but not significantly different in the derived and form priming conditions.

### 3.7 Experiments 5–8: alternative analyses

As for Experiments 1–4, we repeated our analyses with a different data-cleaning protocol and then with MorphoLex frequencies. For Experiments 5–7, the altered data-cleaning protocol resulted in a significant effect of surface frequency for the form priming condition. There were no other changes to the pattern of findings, and switching to MorphoLex frequencies led to no changes. For Experiment 8, both the altered data-cleaning protocol and the switch to MorphoLex frequencies led the previously significant interaction with surface frequency to become non-significant. There were no other changes. Full details are reported in the supplementary materials on OSF.

### 3.8 Experiments 5–8: summary

Experiments 5–8 repeated Experiments 1–4 but presented primes immediately before the targets rather than having them preceding by long distances. We found significant short-lag affix priming (Experiment 5), identity priming (Experiment 6), stem priming (Experiment 7), and derived priming (Experiment 8). We also found significant short-lag semantic and form priming across Experiments 5–8, in contrast to Experiments 1–4, in which semantic and form priming were not evident at long lags. This shows that it was indeed possible for priming to occur with our stimuli.

## 4 Discussion

In this series of eight experiments, we found evidence for long-lag derivational affix priming and identity priming, but not long-lag semantic, form, or derived-to-stem priming. We found weaker evidence for long-lag stem-to-derived priming, as this effect depended on our data-cleaning protocol. In contrast, when the prime immediately preceded the target, we found evidence for all of affix, identity, semantic, form, stem-to-derived, and derived-to-stem priming. We believe the replication of long-lag affix priming between our Experiment 1 and VanWagenen (2014), and the repeated absence of long-lag semantic and form priming in VanWagenen (2014) and our Experiments 1–4, makes these findings even more convincing.

In the long-lag experiments, higher surface frequency of the target always led to faster reaction time, regardless of the type of priming. We found an interaction between prime type and prime distance, but follow-up pairwise contrasts revealed no significant differences between conditions, and there was no main effect of prime distance. Prime distance effects would be expected under a residual activation account of identity/affix priming, but not under a recollection account.

In the short-lag experiments, we observed a significant interaction between condition (prime type) and surface frequency of the target. There was a facilitatory effect of surface frequency when there was no prime (higher frequency associated with faster reaction times, as is generally observed in lexical decision, and as was observed for the long-lag paradigm). This also occurred for the semantic and form priming conditions, as well as in the stem priming condition, to a lesser extent. The surface frequency effect in the no-prime condition was significantly larger than in the affix, identity, and stem priming conditions, but not significantly different from the semantic and form priming conditions.

The weaker evidence for long-lag stem priming (and lack of derived priming) in our experiments – in the face of consistent affix and identity priming – plausibly also reflects the role of context in recollection-memory-based priming. We did not find long-lag stem priming using our planned data-cleaning protocol (identical to VanWagenen (2014)’s) despite the fact that we had 85% power to detect an effect as small as the affix priming effect from VanWagenen (2014), and stem priming can reasonably be expected to have an equal or larger effect size. This effect was only visible with a more conservative protocol. However, in both analyses we did not observe a significant difference between the two conditions. Thus, we cannot make strong conclusions about the presence or absence of a stem priming effect, although stem priming may be somewhat less robust. This deserves follow-up in future work. The suggested fragility of these more standard “morphological” priming conditions does not necessarily constitute failure to replicate results from the literature, for the reasons previewed in the Introduction. First, previous long-lag morphological priming studies employed inflectional affixes, rather than the derivational affixes used here. Second, previous studies repeat the same affixes over and over in the morphological priming conditions, while here each potential suffixed prime or target bore a unique suffix for the experiment. Unfortunately, the design of the present experiment does not allow us to determine which of these factors, or perhaps others, might be relevant.

Nevertheless, we did not predict an asymmetry between affixes and stems in long-lag priming, and we can only speculate as to why they might differ. In long-lag priming, our hypothesis is that the visual form of a morpheme triggers recollection of the occurrence of the same morpheme earlier in the experiment, and reactivation of this representation, speeding up a decision that the target item is a word. A key property of recollection memory is that it is context dependent (Yonelinas 2002). Key contextual features of a stimulus that are regularly used in memory research include which side of a computer screen a word or picture was presented on, or whether a word was spoken in a male or female voice. In the random series of words in our experiments, environment, sentential, or list context has been removed as a possible trigger of recollection. However, affixes and stems have morphological contexts within the word in which they occur. Repetition (identity) priming, affix priming, and long-lag stem priming can be characterized as differing in within-word context. In full repetition priming, the stem appears in the same morphological context in prime and target. For affix and stem priming, the contextual contrast is greater for a single stem occurring with different affixes than for a single affix occurring with different stems. That is, finding *work* first in the context of no suffix or a null suffix, and then in the context of -*er,* is a strong context change, while finding -*er* first in the context of *work* and then in the context of *farm* is a lesser context change. This is why a stem priming condition in which both prime and target are derived forms, with different affixes, would be an interesting comparison. However, as we mention in the Methods section, this would be a challenging design to execute and would probably require repeating affixes, which is undesirable.

Affix frequency, which could matter for within-word context effects, did not significantly contribute to model fit. This suggests that the level of context that matters is not the form level that these variables are defined over, which has been associated with the M170 evoked response in previous research (Lewis, Solomyak and Marantz 2011; Gwilliams, Lewis and Marantz 2016). Variables defined over grammatical properties, such as part of speech continuation entropy (Linzen, Pylkkänen and Marantz 2013), might prove to be better predictors of the strength of the retrieval context. We leave this speculation for further research.

Within linguistics, the affix/stem asymmetry is associated with distinct, competing representational hypotheses. Within Distributed Morphology, for example, selection relations between stems and affixes (which affixes go with which stems) are represented by lists of stems tied to the affix, and the particular interpretation of a stem in the context of an affix is also information associated with the affix, interpreted as a function of the meaning of a stem. In other theories, derivatives of a stem are organized in derivational families of the stem, and the affixed forms of a stem are thus represented with the stem, rather than as information associated with the affix (Schreuder & Baayen 1997). At this general level of competing hypotheses, our results would tend to support the Distributed Morphology approach, since the recognition and interpretation of a stem + suffix combination involves checking the stem on a list associated with the suffix, rather than examining a family of words sharing the same stem. All occurrences of an affix within Distributed Morphology would take one to the same representational space, while each occurrence of a stem in a distinct affixal environment would take one to the representational space of that particular affix, making the affixal context particularly salient.

The hypothesis, then, is that recollection memory over long lags is affected by the similarity of the prime and target contexts. In the previous long-lag stem priming experiments, all prime stems were encountered in a very small set of repeated morphological contexts, which varied minimally from the target contexts. For example, in Kouider and Dupoux (2009), stem primes always occurred in the context of feminine gender inflection, while the targets always occurred with masculine gender inflection. This repeated, and consistent, correspondence between the prime and target context seems to support long-lag priming. In our experiments, no such repetition or consistency occurred for either the affix or stem priming conditions.

Although in broad strokes, an affix/stem asymmetry could be taken to support affix-centered representations of affix/stem dependencies over stem-centered representations, the linguistic literature has explored a wide range of properties that distinguish derivational affixes and types of derivations. That is, if the degree of long-lag affix priming depends on representational properties of affixes, we should be able to make more fine-grained predictions about modulating the priming effect based on distinctions among affixes. For example, we could consider effects of the transparency with which the affix relates to the stem. The difficult nature of this paradigm, where each affix may occur only once or twice per subject, makes such a research program challenging, but these possibilities should be explored in future work. The priming effects that we observed for individual stimuli are available in the supplementary materials on OSF for both the long-lag and the short-lag designs.

## 5 Conclusion

We conducted a series of visual lexical decision priming experiments investigating the extent to which a prime and target which share an English derivational affix *(*-*ish*, -*ize*, etc.) demonstrated the same or different priming effects as other kinds of linguistically related prime/target pairs over long and short lags. We made the case that finding long-lag affix priming would argue that affixes are representational units stored in the mental lexicon, as expected by linguistic theories such as Distributed Morphology, and counter to the expectations of Word and Lexeme Based theories. Our Experiment 1 robustly confirmed this prediction: while semantic and form primes failed to have any effect on the speed of making a lexical decision to their targets, significant priming was observed for primes and targets that shared a derivational affix over lags of as many as 33 intervening items. The magnitude of this effect (29 ms, a ~4% decrease in RTs compared to baseline) is similar to the effects reported for long-lag stem priming in the literature (e.g,. Kouider & Dupoux 2009: an average 4.2% decrease; Marslen-Wilson & Tyler 1997: a decrease of ~5%). Also consistent with this long-lag stem priming literature, the distance between the prime and the target had no effect on the priming magnitude for affix priming, consistent with a recollection memory account of long-lag priming, rather than a spreading activation account.

Note that in this paper we have called “morphemes” suffixes that have a sound + spelling (/ɪti/ + ity), a syntactic consequence (attaches to adjectives to create nouns), and a meaning (the state of being the adjective). Within Distributed Morphology and many other so-called “realizational” or “non-lexical” theories of morphology, the term “morpheme” is restricted to a bundle of syntactic features, which are mapped to phonological/orthographic forms in the course of a syntactic derivation.^10^ The phonological pieces to which morphemes are mapped are called “Vocabulary Items” in DM. We’ve glossed over the distinction between the informal use of “morpheme” to mean the combination of the more technical DM morpheme and a Vocabulary Item since, in the experiments described here, we could not distinguish accounts of the data that claimed that DM morphemes are primed at long lags from accounts that claimed that DM Vocabulary Items, or combinations of morphemes and Vocabulary Items, are primed. Since, as explained above, we also tried to hold the meaning of the derivational suffixes constant in the prime-target pairs, the repetition involved in this long-lag priming included repetition of letters, morphemes (in the DM sense) and meaning.

Our inability to separate the effects of morphemes and Vocabulary Items in these experiments makes it difficult to evaluate the importance of the results for certain psycholinguistic theories of morphological processing. For example, Crepaldi et al. (2016) suggest that suffixes, unlike stems, are not associated with “lemmas” as in the work of Levelt (1993). For comparison with Distributed Morphology, the lemma would be a pairing of a morpheme and a Vocabulary Item. If long-lag priming involves the lemma level, then the experiments conflict with the Crepaldi et al. theory. However, if long-lag priming is restricted to Vocabulary Items, this theory is compatible with the data. In addition, it is difficult to assess the implications of the experiment for certain linguistic theories of morphology, such as A-Morphous Morphology (Anderson 1992). In A-Morphous Morphology, the phonological forms of (many) suffixes aren’t items or “pieces,” but simply the output of processes applying to stems. If all affixes are the output of processes, but all stems involve phonological forms that are “pieces,” the long-lag priming might be used to challenge A-Morphous Morphology, since such priming is seen for both stems and suffixes. However, there are versions of such theories (e.g., versions of Lexical Phonology and Morphology, for example Kiparsky 1982) in which the phonological forms of stems are also the result of rules (for Kiparsky, an “identity” rule), rather than being stored pieces. Such versions would be compatible with our results, since long-lag priming could be said to be sensitive to repetition of a morphophonological rule, for both stem and affix priming, across derivation and inflection.

While derivational affix priming behaved precisely as predicted by Distributed Morphology and other linguistic and psycholinguistic models that treat affixes as morphological units, we did not find strong evidence for long-lag stem priming. However, as discussed above, rather than understanding this as a surprising failure to replicate previous results, we propose that the key differences between our experiments and all other long-lag stem priming experiments suggest an interesting potential asymmetry between the status of stems and derivational affixes as salient contexts for generating memory traces, an asymmetry that speaks to the role of context in recollection memory. This asymmetry, and our interpretation of it, clearly require follow-up research specifically targeting this interpretation, such as neuroimaging research investigating whether the short and long-lag priming effects found in this experiment have distinct neural sources, or experiments manipulating the amount and kind of linguistic context primes are embedded in.

## Figures and Tables

**Table 1:** Stimulus characteristics for targets and primes.

	Targets	Affix primes	Semantic primes	Form primes	Stem primes
Raw surface frequency					
Mean	135.38	171.87	572.56	202.78	1141.49
*SD*	241.71	373.94	969.15	573.08	1530.74
Minimum	2	2	4	1	1
Maximum	1231	1794	4174	3379	4944
Length (characters)					
Mean	8.35	8.65	6.35	6.75	5.35
*SD*	1.61	1.59	1.56	1.15	1.49
Minimum	6	4	3	4	3
Maximum	14	13	9	9	10
Number of syllables					
Mean	2.80	2.80	2.03	2.18	1.65
*SD*	0.72	0.72	0.70	0.50	0.53
Minimum	2	2	1	1	1
Maximum	4	4	4	3	3
Transition probability of suffix					
Mean	0.24	0.15			
*SD*	0.29	0.23			
Minimum	0.00	0.00			
Maximum	0.94	0.87			
LSA similarity between prime and target					
Mean		0.08	0.19	0.06	0.35
*SD*		0.09	0.12	0.08	0.19
Minimum		−0.09	−0.10	−0.06	0.08
Maximum		0.37	0.63	0.29	0.72
Orthographic Levenshtein distance between prime and target					
Mean		4.95	7.38	3.65	3.13
*SD*		1.26	1.33	1.21	0.79
Minimum		2	5	2	1
Maximum		8	11	8	4

**Table 2:** Data pre-processing details for each experiment.

Experiment	1	2	3	4	5	6	7	8
Datasets logged	67	60	123	52	60	67	174	59
Datasets analyzed	57	52	108	46	50	55	153	54
Target accuracy	97.1%	96.2%	95.6%	98.0%	95.6%	94.7%	96.4%	97.4%
Prime accuracy	94.5%	91.5%	93.1%	90.3%	90.1%	88.9%	91.7%	89.2%
Individual RTs excluded	2.3%	2.3%	2.1%	1.9%	1.9%	1.9%	2.2%	1.2%
Observations included per target item	42–57	30–52	59–108	34–46	35–50	36–55	113–153	36–54

**Table 3:** Mean reaction times and accuracy by condition for long-lag priming Experiments 1–4 with VanWagenen (2014) included for comparison.

Experiment	Priming Condition	Mean RT in ms (*SE*)	Priming magnitude	Effect size [95% CI]	Bayes Factor	Accuracy
VW (2014)						
	None	551.6 (9.1)	baseline			
	Affix	531.6 (8.6)	−20	−0.28 [−0.52, −0.033]		
	Form	546.5 (9.0)	−5.1	−0.069 [−0.31, 0.17]		
	Semantic	555.5 (10.0)	−3.9	0.050 [−0.19, 0.29]		
Exp. 1						
	None	742.97 (15.79)	baseline			96.5%
	Affix	713.72 (13.09)	−29.26	−0.35 [−0.62, −0.083]	3.49	97.7%
	Form	732.01 (14.38)	−10.97	−0.099 [−0.36, 0.16]	0.19	97.2%
	Semantic	739.22 (15.26)	−3.75	−0.049 [−0.31, 0.21]	0.15	97.0%
Exp. 2						
	None	706.64 (16.91)	baseline			96.2%
	Identity	655.31 (13.24)	−51.33	−0.76 [−1.08, −0.45]	12297.26	97.9%
	Form	694.47 (14.67)	−12.17	−0.14 [−0.41, 0.14]	0.24	95.6%
	Semantic	695.12 (14.62)	−11.52	−0.12 [−0.40, 0.15]	0.22	95.6%
Exp. 3						
	None	746.55 (14.94)	baseline			95.1%
	Stem	734.76 (13.59)	−11.79	−0.11 [−0.30, 0.080]	0.20	96.1%
	Form	743.42 (15.06)	−3.13	−0.030 [−0.22, 0.16]	0.11	94.9%
	Semantic	744.99 (14.55)	−1.56	−0.016 [−0.21, 0.17]	0.11	96.1%
Exp. 4						
	None	642.32 (15.88)	baseline			97.4%
	Derived	640.11 (17.71)	−2.22	−0.021 [−0.31, 0.27]	0.16	98.5%
	Form	644.60 (16.62)	+ 2.27	0.024 [−0.27, 0.32]	0.16	98.5%
	Semantic	636.97 (16.32)	−5.36	−0.066 [−0.36, 0.23]	0.18	97.8%

**Table 4:** Mean reaction times and accuracy by condition for short-lag priming Experiments 5–8.

Experiment	Priming Condition	Mean RT in ms (*SE*)	Priming magnitude	Effect size [95% CI]	Bayes Factor	Accuracy
Exp. 5						
	None	709.81 (22.34)	baseline			93.2%
	Affix	627.90 (16.15)	−81.90	−0.69 [−1.01, −0.38]	1619.45	97.6%
	Form	674.98 (20.80)	−34.82	−0.34 [−0.63, −0.05]	2.08	94.8%
	Semantic	671.20 (19.18)	−38.60	−0.35 [−0.64, −0.06]	2.44	96.6%
Exp. 6						
	None	738.08 (18.35)	baseline			91.6%
	Identity	634.10 (19.57)	−103.98	−0.79 [−1.10, −0.49]	51714.07	96.5%
	Form	699.25 (21.23)	−38.83	−0.32 [−0.60, −0.05]	1.97	93.8%
	Semantic	695.39 (20.11)	−42.69	−0.50 [−0.78, −0.22]	49.85	96.5%
Exp. 7						
	None	722.25 (10.36)	baseline			94.0%
	Stem	635.72 (7.81)	−86.53	−0.89 [−1.08, −0.70]	9.09e + 17	98.1%
	Form	684.48 (9.46)	−37.76	−0.41 [−0.57, −0.24]	8570.91	96.2%
	Semantic	687.37 (9.56)	−34.87	−0.36 [−0.53, −0.20]	978.47	97.1%
Exp. 8						
	None	648.36 (11.61)	baseline			95.2%
	Derived	584.55 (11.53)	−63.81	−0.91 [−1.24, −0.59]	877083.7	98.9%
	Form	613.61 (13.19)	−34.75	−0.49 [−0.78, −0.21]	37.46	97.0%
	Semantic	616.93 (12.93)	−31.43	−0.50 [−0.78, −0.21]	43.57	99.1%

**Table 5:** Estimated marginal means and pairwise contrasts between priming conditions for Experiments 1–3. The degrees-of-freedom method is asymptotic, so degrees of freedom for both the estimate and the contrast estimate are infinite.

Prime type	Estimate [95% CI]	*SE*	Contrast estimate	*SE*	*z* ratio	*p*
None	701 [674, 729]	14.2				
Affix	671 [639, 704]	16.7	30.07	11.03	2.726	.030 *
Identity	654 [621, 687]	16.9	47.27	11.64	4.062	< .001 *
Stem	687 [657, 717]	15.3	14.36	8.40	1.711	.338
Semantic	696 [668, 724]	14.2	5.27	6.55	0.804	.919
Form	696 [668, 724]	14.3	5.21	6.71	0.776	.929

**Table 6:** Fixed effects for Experiments 1–3, evaluated by likelihood ratio test.

Effect	Estimate	*SE*	χ^2^ (*df*)	*p*
prime type			25.13 (5)	<.001*
surface frequency	−31.52	6.71	17.53 (1)	<.001*
affix frequency	−1.65	5.64	0.08 (1)	.771
transition probability of suffix	4.49	5.94	0.57 (1)	.451
affix length	−7.63	12.99	0.34 (1)	.558
reaction time to previous item	17.12	2.44	48.71 (1)	<.001*
previous item being nonword	18.90	4.72	16.03 (1)	<.001*
accurate on previous item	−2.08	11.16	0.03 (1)	.852
length	9.08	5.22	2.93 (1)	.088
order	−0.15	0.06	6.67 (1)	.010*
list			3.26 (3)	.354
group			2.48 (3)	.479
experiment			5.35 (2)	.069

**Table 7:** Fixed effects for Experiment 4, evaluated by likelihood ratio test.

Effect	Estimate	*SE*	χ^2^ (df)	*p*
prime type			0.99 (3)	.803
surface frequency	−10.67	2.64	13.59 (1)	<.001*
reaction time to previous item	22.80	4.60	24.21 (1)	<.001*
previous item being nonword	19.47	8.96	4.71 (1)	.030*
accurate on previous item	4.21	18.01	0.05 (1)	.815
length	2.80	3.33	0.70 (1)	.401
order	0.22	0.11	4.09 (1)	.043*
list			6.34 (3)	.096
group			9.65 (3)	.022*

**Table 8:** Estimated marginal means and pairwise contrasts between priming conditions for Experiments 5–7. The degrees-of-freedom method is asymptotic, so degrees of freedom for both the estimate and the contrast estimate are infinite.

Prime type	Estimate [95% CI]	*SE*	Contrast estimate	*SE*	*z* ratio	*p*
None	693 [670, 716]	11.8				
Affix	628 [597, 659]	15.8	64.6	12.59	5.131	<.001*
Identity	615 [586, 645]	15.1	77.5	11.68	6.636	<.001*
Stem	629 [604, 654]	12.8	64.0	8.03	7.965	<.001*
Semantic	664 [641, 687]	11.8	29.0	6.60	4.397	<.001*
Form	655 [632, 678]	12.0	37.8	6.75	5.605	<.001*

**Table 9:** Fixed effects for Experiments 5–7 evaluated by likelihood ratio test.

Effect	Estimate	*SE*	χ^2^ (*df*)	*p*
prime type			127.77 (5)	<.001*
surface frequency	−31.72	6.52	19.26 (1)	<.001*
affix frequency	2.33	5.13	0.21 (1)	.650
transition probability of suffix	1.40	5.43	0.07 (1)	.797
affix length	−3.01	11.84	0.06 (1)	.800
reaction time to previous item	32.04	2.08	230.18 (1)	<.001*
length	8.19	4.76	2.85 (1)	.091
order	−0.16	0.05	11.58 (1)	<.001*
list			7.02 (3)	.071
group			4.15 (3)	.246
experiment			1.56 (2)	.458
prime type × surface frequency			39.81 (5)	<.001*

**Table 10:** Estimated marginal trends and pairwise contrasts between the surface frequency effect in each priming condition for Experiments 5–7. The degrees-of-freedom method is asymptotic, so degrees of freedom for both the estimate and the contrast estimate are infinite.

Prime type	Estimate [95% CI]	*SE*	Contrast estimate	*SE*	*z* ratio	*p*
None	−31.72 [−44.5, −18.93]	6.52				
Affix	−13.01 [−29.6, 3.58]	8.46	−18.71	6.49	−2.882	.019*
Identity	−6.14 [−22.1, 9.85]	8.16	−25.57	6.11	−4.185	<.001*
Stem	−17.79 [−31.3, −4.28]	6.89	−13.92	4.26	−3.266	.005*
Semantic	−29.95 [−42.7, −17.16]	6.53	−1.77	3.70	−0.477	.991
Form	−35.15 [−48.1, −22.24]	6.59	3.43	3.76	0.912	.867

**Table 11:** Estimated marginal means and pairwise contrasts between priming conditions for Experiment 8. The degrees-of-freedom method is Kenward-Roger.

Prime type	Estimate [95% CI]	*SE*	*df*	Contrast estimate	*SE*	*df*	*t* ratio	*p*
None	642 [615, 670]	14.0	133					
Derived	574 [546, 602]	14.2	139	68.3	12.0	1887	5.708	<.001*
Semantic	598 [569, 626]	14.2	140	44.8	11.8	1876	3.802	<.001*
Form	595 [566, 624]	14.6	154	47.3	12.5	1895	3.798	<.001*

**Table 12:** Fixed effects for Experiment 8, evaluated by likelihood ratio test.

Effect	Estimate	*SE*	χ^2^ (*df*)	*p*
prime type			13.04 (3)	.005*
surface frequency	−7.61	2.25	4.53 (1)	.033*
reaction time to previous item	18.21	3.78	23.49 (1)	<.001*
length	14.57	3.40	7.25 (1)	.007*
order	−0.35	0.09	14.10 (1)	<.001*
list			3.58 (3)	.311
group			1.69 (3)	.640
prime type × surface frequency			8.83 (3)	.032*

**Table 13:** Estimated marginal trends and pairwise contrasts between the surface frequency effect in each priming condition for Experiment 8. The degrees-of-freedom method is Kenward-Roger.

Prime type	Estimate [95% CI]	*SE*	*df*	Contrast estimate	*SE*	*df*	*z* ratio	*p*
None	−9.04 [−17.6, −0.462]	4.36	356					
Derived	−8.74 [−17.1, −0.429]	4.22	295	−0.302	5.56	1896	−0.054	1.000
Semantic	−23.16 [−31.9, −14.39]	4.46	360	14.12	5.67	1888	2.490	.035*
Form	−16.06 [−24.6, −7.49]	4.36	333	7.02	5.66	1894	1.241	.459

## Data Availability

All data and analysis code, complete model outputs, and supplementary materials including stimulus lists, stimulus characteristics, details for alternative analyses, by item observation counts, and by item priming magnitudes are available on the Open Science Framework (OSF) at the following URL: https://osf.io/v42a3/.

## Footnotes

1 The terms “stem,” “lemma,” “base,” and “root” are used variously by different researchers and subfields to describe the *teach* part of *teacher* and *teaches*. We have generally opted to use “stem” throughout this manuscript as the most theory-neutral term.

2 Since the popularization of Pinker’s Words and Rules approach to complex word recognition (Pinker 1998), the status of affixes has been widely debated in the psycholinguistic literature in terms of a potential “dual-route” processing system for affixed words. Within Pinker’s approach, what for a linguist are morphologically complex words may be recognized either via decomposition into morphemes (e.g., *walked* = *walk* + -*ed*) or as whole words (e.g., irregular past tense *sank*), depending on factors related to the regularity of the “rule” that combines the relevant morphemes. Two strands of literature contested the proposal that there are “dual routes” to complex word recognition. On the one hand, a literature associated with Rumelhart and McClelland’s (1986) Connectionist approach to word recognition claimed that all words are recognized as wholes, with morphemes emerging as statistical generalizations within connectionist systems that relate phonological, orthographic, and semantic properties of words. On the other hand, a literature associated with Distributed Morphology (DM) (Halle & Marantz 1993) – and, more generally, with traditional linguistic analysis – claimed that all words are decomposed, including “irregular” past tenses and derived words with relatively opaque meanings. Both approaches denied that there is a categorical difference between composed structures, including complex words, and memorized structures, allowing that experience with language involves learning statistical regularities about complex, as well as atomic, linguistic units (Marantz 2013; Fruchter, Stockall & Marantz 2013).

3 We use here the term employed at the time of the study to describe the Bosnian/Croatian/Serbian macrolanguage which was then an official language of Yugoslavia.

4 The code for the lexical decision task was loosely adapted from a template created by Brian Dillon.

5 https://spellout.net/ibexfarm.

6 Effect sizes were calculated using function cohen.d in package effsize (Torchiano 2018).

7 Bayes Factors were calculated using function ttestBF in package BayesFactor (Morey & Rouder 2018).

8 Regression models were evaluated using function *mixed* from the *afex* package (Singmann et al. 2021).

9 Follow-up tests were conducted using function *contrast* from the *emmeans* package (Lenth 2021).

10 Recently, Milin et al. (2017) have described an approach to word recognition that both denies morpheme status to suffixes and avoids the standard Connectionist move of encoding morpheme units in either the phonological or semantic representations that underpin their Discrimination Learning (DL) model. Within this model, the abstract units are “pointers” to vectors in semantic space, rather than being themselves unique vectors in the space. These pointers are called “lexomes,” and language learning is conceptualized as the acquisition of mappings between sound and meaning mediated by lexomes. If we equate the morphemes of Distributed Morphology with the lexomes of Discrimination Learning on the grounds that both are the abstract, atomic units that mediate the connection between sound and meaning, support in this paper for derivational affixes being DM morphemes would translate to support for derivational affixes being lexomes within the DL models.
